# Improving the Preoperative Diagnostic Accuracy of Acute Appendicitis. Can Fecal Calprotectin Be Helpful?

**DOI:** 10.1371/journal.pone.0168769

**Published:** 2016-12-29

**Authors:** Peter C. Ambe, Valerie Orth, Daniel Gödde, Hubert Zirngibl

**Affiliations:** 1 Department of Surgery HELIOS Universitätsklinikum Wuppertal Witten–Herdecke University Heusnerstr. Wuppertal, Germany; 2 Institute of Pathology and Molecular Pathology HELIOS Universitätsklinikum Wuppertal Witten–Herdecke University Heusnerstr. Wuppertal, Germany; University Hospital Llandough, UNITED KINGDOM

## Abstract

**Background:**

Is the patient really suffering from acute appendicitis? Right lower quadrant pain is the most common sign of acute appendicitis. However, many other bowels pathologies might mimic acute appendicitis. Due to fear of the consequences of delayed or missed diagnosis, the indication for emergency appendectomy is liberally made. This has been shown to be associated with high rates of negative appendectomy with risk of potentially serious or lethal complications. Thus there is need for a better preoperative screening of patients with suspected appendicitis.

**Methods:**

This prospective single center single-blinded pilot study was conducted in the Department of surgery at the HELIOS Universitätsklinikum Wuppertal, Germany. Calprotectin was measured in pre-therapeutic stool samples of patients presenting in the emergency department with pain to the right lower quadrant. Fecal calprotectin (FC) values were analyzed using commercially available ELISA kits. Cut-off values for FC were studied using the receiver-operator characteristic (ROC) curve. The Area under the curve (AUC) was reported for each ROC curve.

**Results:**

The mean FC value was 51.4 ± 118.8 μg/g in patients with AA, 320.9 ± 416.6 μg/g in patients with infectious enteritis and 24.8 ± 27.4 μg/g in the control group. ROC curve showed a close to 80% specificity and sensitivity of FC for AA at a cut-off value of 51 μg/g, AUC = 0.7. The sensitivity of FC at this cut-off value is zero for enteritis with a specificity of 35%.

**Conclusion:**

Fecal calprotectin could be helpful in screening patients with pain to the right lower quadrant for the presence of acute appendicitis or infectious enteritis with the aim of facilitating clinical decision-making and reducing the rate of negative appendectomy.

## Background

Acute appendicitis (AA) is a common cause for a visit to the emergency department and appendectomy represents the most commonly performed emergency procedure in surgery [1]. AA is heralded by pain to the right lower quadrant. This might be accompanied by nausea, vomiting and signs of systematic inflammatory response like fever and chills. Besides, blood chemistry might indicate elevated acute phase proteins like C—reactive protein (CRP) and high white blood count (WBC) [2, 3]. These findings are however not specific for AA. In fact, pain to the right lower quadrant with systemic signs of inflammation and elevated inflammatory markers in blood might be due to quiet a handful of pathologies [4, 5]. Especially bowel pathologies including right sided colitis, ileitis or gastroenteritis might present with similar signs and symptoms thus mimicking AA [6, 7]. The spectrum of possible differential diagnosis even gets wider in female patients in reproductive age. The dilemma associated with the diagnosis of AA still prevails despite the extensive use of clinical scoring systems and modern imaging modalities. Because of fear of the consequences of delayed or missed diagnosis, the indication for surgery for suspected AA is lavishly made. It is there not surprising that high rates of negative appendectomy have been reported in literature [8–10].

Calprotectin (Cal) is a 36-kDa heterodimer that belongs to the family of calcium-binding proteins [11]. Cal has been identified as an antimicrobial protein and constitutes about 60% of cytosolic proteins in neutrophil granulocytes [12]. It is secreted into the intestinal lumen during the early phases of intestinal mucosal damage [13, 14]. Cal has been shown to be relatively robust against bacterial degradation at room temperature. This in association with the non-invasive means of sample collection makes Cal an attractive biomarker. Currently, fecal calprotectin (FC) has been shown to be a valuable diagnostic marker for a series of bowel pathologies, e.g. chronic inflammatory bowel diseases [15–17].

Since AA primarily begins at the level of the mucosa, it is thinkable that FC could have a diagnostic value in patients with suspected AA. This hypothesis was tested in a qualitative analysis using calprotectin specific antibodies. Strong immunostainings were recorded in specimens from patients with AA while no reaction was seen in control specimens without AA [18]. The aim of the present study was to investigate the expression of Cal in stool of patients presenting with suspected AA due to pain to the right lower quadrant. We hypothesized that FC would be higher in patients with infectious enteritis compared to those with AA, while patients with AA would have higher FC values in comparison with healthy controls.

## Materials and Methods

This single—center, single–blinded pilot study was conducted at the Department of Surgery, HELIOS Universitätsklinikum Wuppertal, Witten—Herdecke University, Germany. Ethical approval for this study was received from the ethics commission at the Witten–Herdecke University in Germany. The study was conducted in accordance with the ethical principles of the Declaration of Helsinki and the principles of Good Clinical Practice [19]. A written consent was obtained from all patients prior to inclusion in the study. Patients were recruited following presentation in the emergency department with pain to the right lower quadrant and suspected appendicitis. Each patient was seen by an experienced member of the surgical team. The decision to perform emergency / urgent laparoscopy was made after considering findings from patient´s history, physical examination, blood chemistry and abdominal ultrasound sonography, which were performed in all cases. Occasionally, computed tomography was ordered.

### Inclusion and exclusion criteria

All patients 16 years and above with suspected appendicitis were eligible for this pilot study. A written consent was received from each patient or their legal representatives prior to inclusion in the study. Refusal to consent, patient under immunosuppressive treatment, history of inflammatory bowel disease, history of appendectomy, current antibiotic treatment, known gastrointestinal infections and patients on proton pump inhibitors (PPI) were excluded from the study.

### Sample collection

Stool samples were collected prior to treatment initiation. Our institutional standard requires patients to empty their bowel and bladder prior to surgery. Stool samples from surgical candidates were collected at this time. Stool samples from patients admitted for “watchful waiting” were collected during the next bowel movement. All samples were frozen at -80°C prior to analysis.

### Patient care

A standard three-port laparoscopic appendectomy was performed in all cases. Surgery began with an infra-umbilical incision for the placement of the camera port. After pneumoperitoneum was instilled two more ports were inserted; 5 mm in the right lower abdomen and 12 mm in the left lower abdomen under direct visualization. Appendectomy was performed using an endoscopic lineal stapler in all cases. The resected vermiform appendix was removed using an endoscopic retrieval bag. Drains were placed as needed. A single shot antibiotic was given at the beginning of surgery and postoperative antibiotics were continued as needed. All patients managed with “watchful waiting” were closely following via serial clinical examinations, blood chemistry and stool cultures were ordered to role out infectious enteritis. Patients without clinical diagnosis of either AA or enteritis were included in the control group.

### Histopathology and immunochemistry

All surgically removed vermiform appendix were histopathologically examined to confirm the diagnosis. Inflammatory changes of the specimens were appraised on hematoxylin and eosin (HE) stained sections.

**Fig 1:**
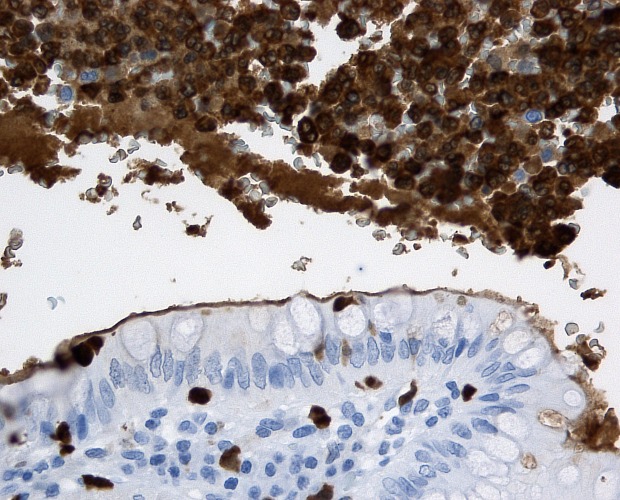
Positive histochemistry with specific anti calprotectin antibody. Positive (gold) immunohistochemical staining (red arrows) with specific anti-calprotectin antibody.

**Fig 2:**
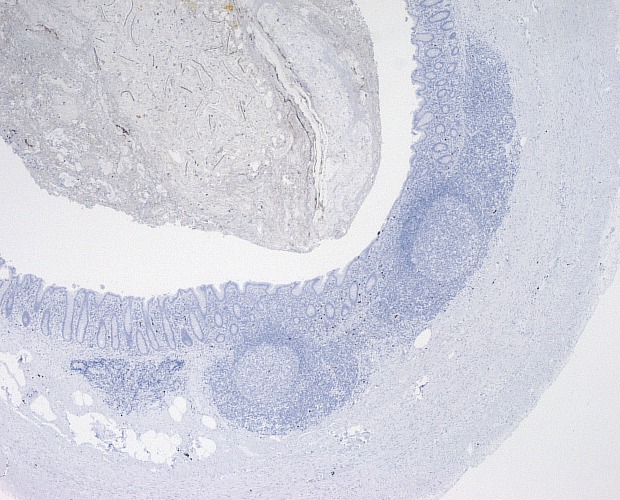
Negative control with specific anti calprotectin antibody. Negative reaction (no gold stain) following immunochemical staining.

The expression of calprotectin in the appendix specimens was assessed by immunohistochemistry using the DAKO Autostainer plus (DakoCytomation) following the manufacturer´s instructions as reported previously [18]: 3–5 μm sections of formalin-fixed, paraffin-embedded tissue was dried overnight at 37°C and deparaffinized. Antigen retrieval was performed using the Target Retrieval Solution (Citrate pH 6,1, 10x, DakoCytomation, cat.no. S1699) after which the slides were steamed for 30 min. Endogenous peroxidases were blocked by incubation with Peroxidase-Blocking Solution (DAKO REAL ^TM^ Peroxidase-Blocking Solution, cat.no. S2023) for 5 minutes. Immunostaining for calprotectin was achieved using calprotectin monoclonal mouse antibodies (Thermo Scientific, Clone MAC 387, cat.no. MA5-12213). The specimens were incubated for 30 min with calprotectin-specific primary antibody (dilution 1:500) followed by subsequent incubations with a visualization reagent based on a dextran technology (EnVision + Dual Link System-HRP, DAKO, cat.no. K4061). The EnVision reagent consists of both secondary rabbit anti-mouse antibody molecules and horseradish peroxidase molecules linked to a common dextran polymer backbone, thus eliminating the need for sequential application of link antibody and peroxidase conjugate. Staining was completed by incubation with a substrate-chromogen (Liquid DAB + Substrate Chromogen System, Dako Cytomation, cat.no. K3468) for 2 x 5 minutes. Enzymatic conversion of the sub-sequentially added chromogen resulted in the formation of a visible brown reaction product at the antigen site. In addition, the nuclei were counterstained with Mayer´s Hematoxylin for 2 min and sealed with coverslips. Immunohistochemical activity was determined in epithelial and inflammatory cells in consideration of the amount of inflammatory cells within the lumen of the vermiform appendix. As reported previously, specimens with confirmed appendicitis stained positive, **Fig 1**, while control specimens without appendicitis (from patients following right hemicolectomy) stained negative, **Fig 2** [18].

### Fecal calprotectin ELISA

To determine the FC concentration, a colorimetric sandwich ELISA (Demeditec Diagnostics, Kiel, Germany) was performed on stool samples according to the manufacturer’s instructions. In brief, diluted standards and unknown samples were added in duplicates and incubated for one hour with shaking. After washing, plates were incubated with HRP-conjugated secondary antibody for 45 minutes. Plates were washed again, and incubated in TMB substrate solution for 8 minutes. Stop solution (2N H_2_SO_4_) was then added to discontinue HRP activity. Absorbances were measured on a plate reader (GloMax, Promega GmbH, Mannheim, Germany) at a wavelength of 450 nm.

### Statistical analysis

The Statistical Package for Social Science (SPSS®), IBM, version 23 was employed to analyze the collected data. Continuous data were first reported using means and standard deviations (±). The mean values for Cal were employed as cut-off values to generate receiver-operator characteristic (ROC) curves. The Area under the curve (AUC) was reported for each ROC curve. Furthermore, ROC curves were generated to analyze the predictive accuracy of FC, WBC and CRP in patients with AA. Besides, ROC curves were used to compare the sensitivity and specificity of WBC and CRP in the diagnosis of AA.

## Results

Stool samples from 32 (17 females and 15 males) patients were available for analysis.

**Fig 3:**
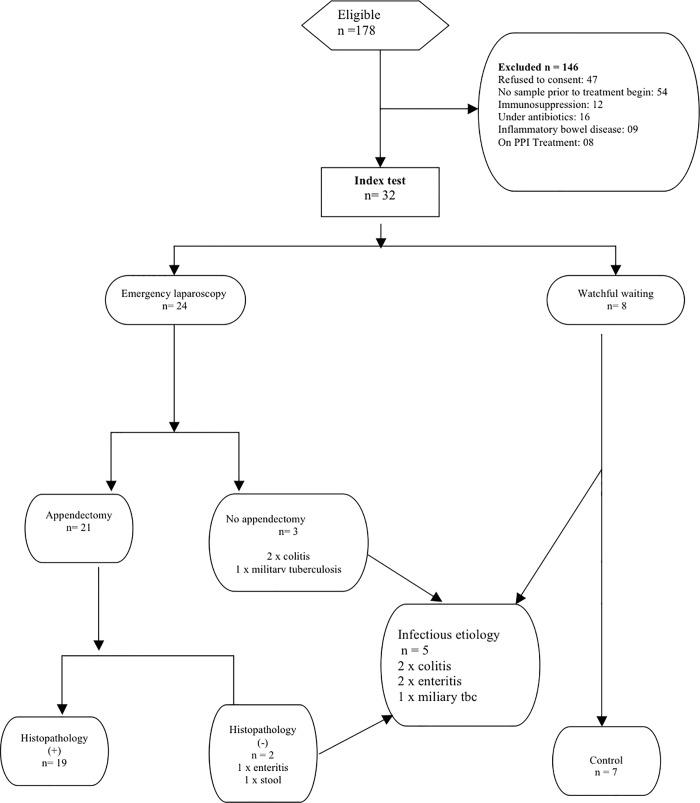
Distribution of the study population. Distribution of the study population according to the STARD Guidelines

The mean age of the study population was 33.7 ± 17.6 years (range 16–87 yrs). Emergency or urgent laparoscopy was performed in 24 cases, leading to appendectomy in 21 cases. Eight patients were conservatively managed with “watchful waiting”. Acute appendicitis was confirmed on histopathology in 19 cases. Histopathology failed to confirm an inflammation in two cases (9.5%) following appendectomy including one case with enteritis and another with stool impaction. In two cases surgery was limited to diagnostic laparoscopy following intraoperative diagnosis of colitis. In the last case, emergency laparoscopy revealed miliary tuberculosis of the peritoneum. **Fig 3** shows the distribution of the study population according to the STARD guidelines for reporting studies of diagnostic accuracy [20].

**Fig 4:**
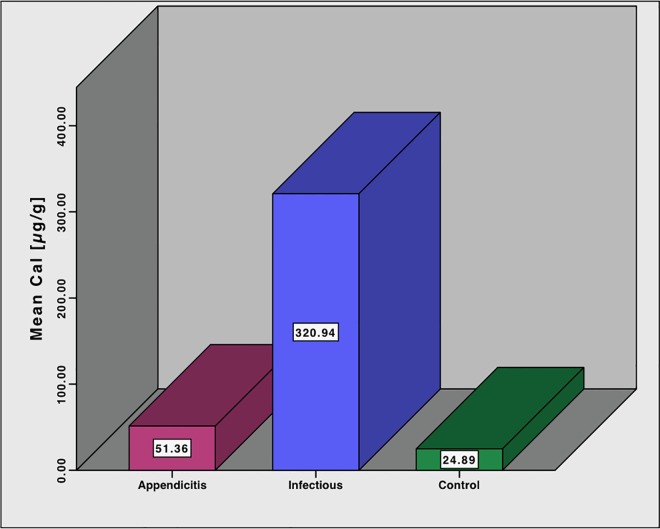
Mean fecal calprotectin values. Mean fecal calprotectin values in the groups with appendicitis, infectious enteritis and healthy controls.

The mean FC value was 51.4 ± 118.8 μg/g in patients with AA, 320.9 ± 416.6 μg/g in patients with gastroenteritis and 24.8 ± 27.4 μg/g in the control group, **Fig 4**. The mean FC values was significantly higher in the group with enteritis compared to the group with AA, p = 0.007. However, no statistically significant difference was seen between the group with AA and the control, p = 0.54.

The mean WBC was 13.2 ± 3.3/μl in patients with AA, 10.9 ± 3.6/μl in patients with gastroenteritis. and 8.5 ± 4.7/μl in the control group. There was no statistically significant difference amongst patients with AA and enteritis with regard to WBC, p = 0.20. However, elevated WBC was recorded significantly more often in the group with AA compared to the control group, p = 0.02.

The mean CRP was 5.3 ± 11.4 mg/dl in patients with AA, 10.6 ± 6.8 mg/dl in patients with gastroenteritis and 0.6 ± 0.9 mg/dl in the control group. The mean CRP was significantly higher in the group with enteritis compared to the AA group, p = 0.03. No statistically significant difference was recorded between the group with AA and the control with regard to CRP, p = 0.06).

**Fig 5:**
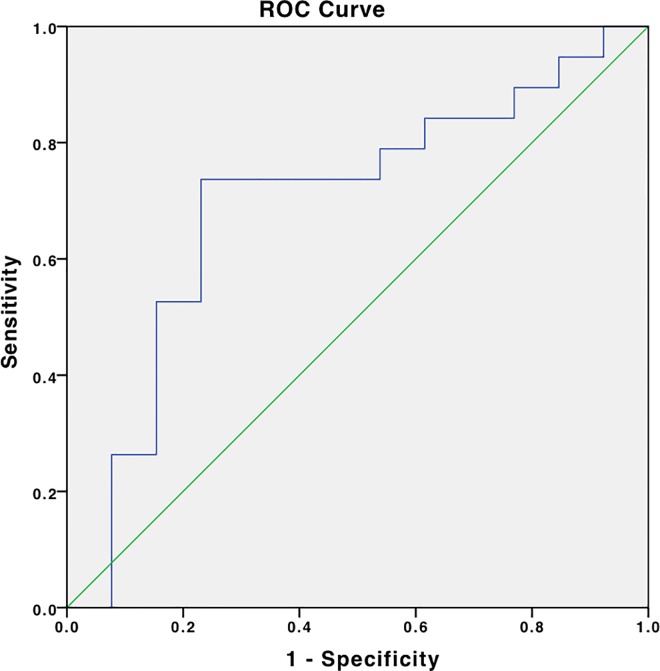
Receiver operating characteristic (ROC) curve for fecal calprotectin. Receiver operating characteristic (ROC) curve for fecal calprotectin in prediction the presence of acute appendicitis. The area under the curve (AUC) was 0.869 (95% confidence interval (CI) CI 0.715–1.0), p = 0.009.

**Fig 6:**
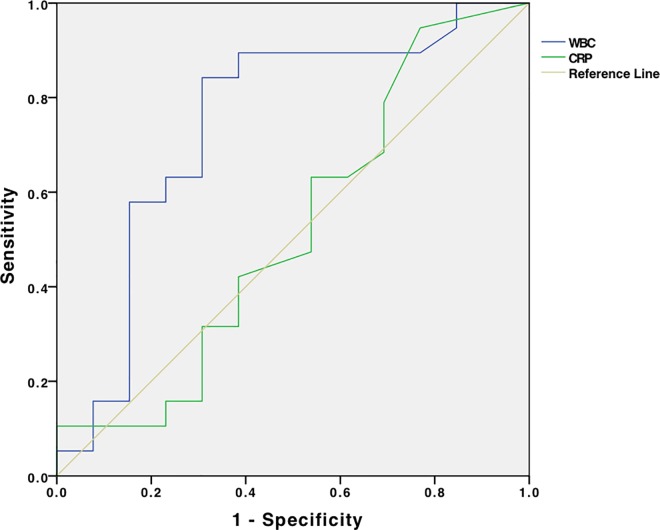
Receiver operating characteristic (ROC) curve for WBC and CRP. Receiver operating characteristic (ROC) curve for WBC (AUC: 0.728, 95% CI: 0.473–0.983, p = 0.098) and CRP in prediction the presence of acute appendicitis (AUC: 0.316, 95% CI: 0.033–0.593, p = 0.181).

ROC curve showed a close to 80% sensitivity and a close to 80% specificity of FC for AA at a cutoff value of 51 μg/g, AUC = 0.86, **Fig 5**. The sensitivity of FC at this cut-off value is 20% for enteritis with a specificity of 25% (AUC:0.110). Similar ROC analysis for WBC showed a sensitivity of about 70% and a specificity of about 65% at a cutoff value of about 11.700/μl, AUC = 0.728, **Fig 6**. ROC analysis of healthy controls at a cut-off value of 24 μg/g indicated a sensitivity of less than 60% with a specificity of about 35% in healthy controls (AUC: 0.537).

## Discussion

Acute appendicitis is a very common reason for a visit to the emergency department. Patients usually present with pain to the right lower quadrant. Fever and chills might be present as signs of systemic involvement. These symptoms however are not specific for AA. Similar complaints might be secondary to a wide variety of pathologies. Infectious gastrointestinal pathologies might present with the same symptoms as AA and therefore represent the most common differential diagnosis of AA. Due to fear of complications secondary to delayed or missed diagnosis, the indication for emergency surgery is liberally made leading to high rates of negative appendectomy. Considering that (negative) appendectomy might be associated with serious and event fatal complications, there is need for a better preoperative screening of patients with suspected appendicitis.

This study investigated the expression of Cal in the stool of patients with suspected appendicitis. Higher Cal values were recorded in patients with infectious enteritis compared to patients with AA. This finding suggests that FC could be a helpful tool in discriminating patients with infectious enteritis from those with appendicitis. The mean FC value was higher in patients with AA compared to healthy controls. ROC analysis revealed a statistically significant diagnostic value of FC at a cut—off value of 54 μg/g with a close to 85% sensitivity and 70% specificity in patients with histopathologically confirmed appendicitis. At this cut-off value, the sensitivity for enteritis was about 20% with a specificity of just 15%. This rather intriguing finding argues for the diagnostic value of FC in patients with suspected appendicitis and is in accordance with our null hypothesis.

Fecal calprotectin is an abundant cytosolic protein of neutrophil granulocytes and represents a well-established biomarker for gastrointestinal pathologies. The expression of FC has been shown to correlate with disease activity on patients with Crohn´s disease [21, 22]. As with other pathologies of the gastrointestinal tract, appendicitis is primarily a mucosal pathology. It is therefore logical, that an inflammation of the vermiform appendix might be associated with changes in the expression of calprotectin. The transfer of inflammatory cells via the lumen of the vermiform appendix into the colonic lumen makes it possible to measure Cal in stool of patients with suspected appendicitis.

In a previous paper, the qualitative expression of Cal in appendectomy specimens was examined via immunohistochemistry using specific anti-calprotectin antibodies [18]. Strong reactions were seen in specimens with proven appendicitis following histopathology, while no reaction was observed in non-inflamed vermiform appendix specimens from patients following right sided hemicolectomy.

The results of the present study strongly support our null hypothesis by confirming higher FC values in patients with infectious conditions compared to those with AA. Equally, the higher levels of Cal in stool of patients with AA compared to the control group is in accordance with our null hypothesis.

An interesting finding in this study is the high sensitivity and specificity of both WBC and FC in patients with histologically confirmed appendicitis. This finding does not only suggest FC as a potential diagnostic marker for appendicitis but also suggests that the diagnostic accuracy of AA might be improved via a combination of both tests (FC and WBC).

The Cal values recorded in the different groups in this study are clearly related with the area of the mucosa surface involved in the inflammatory process. The surface area of the mucosa involved is infectious enteritis is expected to be larger compared to the surface of the inflamed vermiform appendix. Therefore, higher Cal values are to be expected in cases with infectious enteritis. This argument is also true for the Cal values recorded in patients with appendicitis compared to control (without signs of appendicitis or enteritis).

Calprotectin expression during AA is not limited to the bowel mucosa. In a recently published article by Cikot et al. [23] serum Cal was used to differentiate complicated from uncomplicated appendicitis. Currently, both serum and fecal calprotectin could be quickly analyzed using commercially available point of care units [24, 25]. This opens up room for a quick analysis in the emergency department without waste of time. Thus the measurement of both serum and fecal Cal might be helpful in clinical decision—making in the emergency department. This would not only improve the preoperative diagnosis but might also lead to a more individualized patient care.

Although the small size of the study population is a limitation to the findings recorded in this pilot study, the measurement of Cal in the stool of patients with pain to the right lower quadrant might be helpful in discriminating between patients needing emergency surgery for acute appendicitis and candidates for conservative management. However, further investigation would be needed to establish the diagnostic value of both serum and fecal Cal in patients with suspected appendicitis. Equally, the possible diagnostic value of FC in distinguishing patients with AA from those with non-infectious causes of right lower quadrant pain is still to be investigated. Our study group is currently initiating an investigation with the aim of studying the additive effect of serum and fecal Cal on the diagnostic accuracy of appendicitis. We would be happy to present our results in the nearest future.

Taken together, our results suggest FC as a potential marker for screening patients with pain to the right lower quadrant with regard to the present of acute appendicitis or infectious bowel pathologies. Translating the findings from this study into clinical practice could have a huge impact management by facilitating clinical decision-making with regard to the need of an emergency appendectomy.

## Conclusion

Fecal calprotectin could be helpful in screening patients with pain to the right lower quadrant for the presence of acute appendicitis or infectious enteritis with the aim of facilitating clinical decision-making and reducing the rate of negative appendectomy.

## Abbreviations

AA: Acute Appendicitis
FC: Fecal Calprotectin
Cal: Calprotectin
CRP: C- Reactive Protein
ROC: Receiver—Operator Characteristics
WBC: White Blood Count
